# Whole-Genome Sequencing and Molecular Analysis of Ceftazidime–Avibactam-Resistant KPC-Producing *Klebsiella pneumoniae* from Intestinal Colonization in Elderly Patients

**DOI:** 10.3390/antibiotics12081282

**Published:** 2023-08-03

**Authors:** Giulia Errico, Maria Del Grosso, Michela Pagnotta, Manuela Marra, Maria Carollo, Marina Cerquetti, Elena Fogato, Elisabetta Cesana, Flaminia Gentiloni Silverj, Dorjan Zabzuni, Angelo Rossini, Annalisa Pantosti, Marco Tinelli, Monica Monaco, Maria Giufrè

**Affiliations:** 1 Department of Infectious Diseases, Istituto Superiore di Sanità, 00161 Rome, Italy; 2 Core Facilities Technical-Scientific Service (FAST), Istituto Superiore di Sanità, 00161 Rome, Italy; 3 Laboratory of Clinical Microbiology, ASP ‘Golgi-Redaelli’, 20146 Milan, Italy; 4 IRCCS Istituto Auxologico Italiano, San Luca Hospital, 20149 Milan, Italy; 5 Medical Direction, Fondazione IRCCS, Ca’ Granda Ospedale Maggiore Policlinico, 20122 Milan, Italy; 6 IRCCS Fondazione Santa Lucia, 00179 Rome, Italy

**Keywords:** carbapenem-resistant Enterobacterales, ceftazidime–avibactam, *Klebsiella pneumoniae*, colonization

## Abstract

Ceftazidime–avibactam (CAZ-AVI) is an active antibiotic combination of a β-lactam–β-lactamase inhibitor against carbapenemase-producing Enterobacterales. Reports of resistance to CAZ-AVI other than metallo-β-lactamases have increased in recent years. The aim of this study was to analyze KPC-*Klebsiella pneumoniae* (KP) isolates resistant to CAZ-AVI from the intestinal carriage of hospitalized elderly patients in Italy, in February 2018–January 2020. Characterization of CAZ-AVI-resistant KP isolates, including MLST, resistome, virulome and plasmid content, was performed by WGS analysis. Out of six CAZ-AVI-resistant KP isolates, three belonged to ST101 and three to ST512; two isolates produced KPC-3 (both ST512), four had mutated KPC-3 (KPC-31, in ST101 and ST512, and KPC-46, both ST101). All CAZ-AVI-resistant KP isolates were multidrug-resistant and carried several resistance genes. The yersiniabactin *ybt*9 gene cluster was present in all ST101 isolates, while, in ST512 isolates, no virulence genes were detected. Several plasmids were detected: IncF was present in all isolates, as well as IncR and Col440 in ST101 and IncX3 in ST512 isolates. In conclusion, it is important to monitor the circulation of *K. pneumoniae* resistant to CAZ-AVI to prevent the spread of clones causing difficult-to-treat infections. The presence of mutated KPC-3 in high-risk *K. pneumoniae* clones resistant to CAZ-AVI in hospitalized patients deserves attention.

## 1. Introduction

The spread of carbapenemase-resistant *K. pneumoniae* (CR-KP) represents a global threat to public health, causing nosocomial outbreaks and having limited therapeutic options [1,2]. Recently, infections caused by CR-KP have increased worldwide and are associated with high morbidity and mortality rates [3]. Italy is one of the European countries with the highest percentage of carbapenem-resistance in *K. pneumoniae* isolates, and carbapenemase production is the most common resistant mechanism, with KPC being the most frequently detected enzyme [4]. A few antibiotics are active in treating infections caused by carbapenemase-producing *K. pneumoniae* (CP-KP), such as tigecycline and colistin [5,6]. Nevertheless, resistance to colistin and tigecycline has been reported and problems of toxicity have been a major issue in using such antibiotics [7]. Novel β-lactam/β lactamase inhibitor combinations (BLICs), such as ceftazidime–avibactam (CAZ-AVI), meropenem–vaborbactam and imipenem–relebactam, were introduced a few years ago and represent a valid alternative to last-resort antibiotics [8,9]. These combinations are active against class A and class C β-lactamases but have no activity against metallo-β-lactamases; CAZ-AVI is also active on OXA-48-like β-lactamases [10]. However, soon after their introduction, resistance to these new molecules was increasingly reported, particularly resistance to CAZ-AVI in KPC-producing *K. pneumoniae* (KPC-KP) strains [11]. Although resistance to CAZ-AVI may be due to several mechanisms, including alterations of Ompk35 and Ompk36 porins [12], modifications within the Ω loop of KPC carbapenemase represent the most common mechanism [13,14].

Hospitalized elderly patients are prone to infections caused by pathogens resistant to several antibiotics, including new drug combinations such as CAZ-AVI, particularly if they have multiple comorbidities [15]. In this population, colonization can persist for a long period, as we observed in a previous study [16]; it canwidespread and trigger outbreaks, and the infections are sometimes life-threatening and difficult to control, especially in LTCF [17,18]. In the present study, we report the results of the characterization of KPC-KP resistant to CAZ-AVI obtained in the framework of our previous study on CPE colonization and persistence in the elderly [16].

## 2. Results

### 2.1. Patients Colonized by KPC-KP Resistant to CAZ-AVI and KPC-KP Isolates

**Figure 1:**
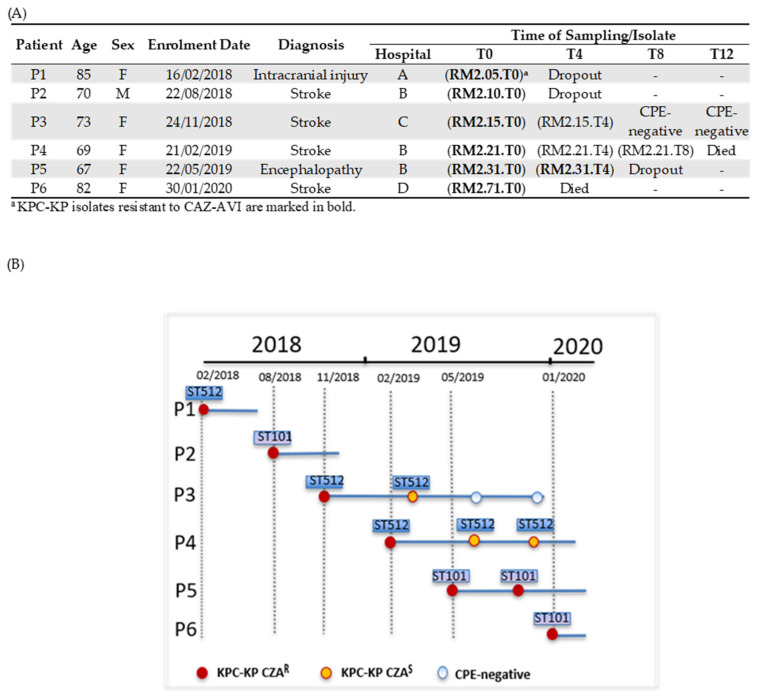
(**A**) Epidemiological data of six patients colonized by KPC-KP resistant to CAZ-AVI, time of sampling and additional KPC-KP isolates obtained at follow-up. (**B**) Timeline of KPC-KP-positive cultures obtained from six patients. Isolation of KPC-KP is indicated by a circle in different colors, according to the isolate type and resistance characteristics. Resistance to CAZ-AVI is indicated by a red circle, while susceptibility to CAZ-AVI is indicated by a yellow circle. A white circle indicates no isolation of CPE. A continuous line indicates the length of stay in the neuro-rehabilitation structure.

In the period of February 2018–January 2020, we collected 112 KPC-KP non-duplicated isolates from the intestinal carriage in elderly hospitalized patients enrolled at hospital discharge (T0) (one isolate per patient) [16]. Out of these KPC-KP isolates, six (5.4%) were resistant to CAZ-AVI (MIC ≥ 16/4 mg/L) and were isolated from six patients (Figure 1A). The six patients (age 67–85 years, five female and one male) were enrolled at discharge from four different hospitals and transferred to the same neuro-rehabilitation structure in Rome, in different time periods (Figure 1A). Two were previously treated with CAZ-AVI (patients 4 and 5).

During the follow-up, at month 4 (T4), two patients were lost (patients 1 and 2), one died (patient 6) and three were still colonized by KPC-KP (patients 3, 4 and 5) (Figure 1A); at month 8 (T8), only patient 4 was still colonized (Figure 1A,B). Four additional KPC-KP isolates were obtained from these three patients during the follow-up: two patients were colonized by KPC-KP susceptible to CAZ-AVI (patients 3 and 4), while one was still colonized by KPC-KP resistant to CAZ-AVI (patient 5) (Figure 1A,B). This patient received treatment with CAZ-AVI for bacteremia due to CR-KP during the follow-up period. Overall, a total of ten KPC-KP isolates were further studied: seven KPC-KP resistant to CAZ-AVI and three KPC-KP susceptible to CAZ-AVI (Supplementary Table S1).

### 2.2. Whole-Genome Sequencing and In Silico Analysis 

WGS analysis of the six KPC-KP isolates resistant to CAZ-AVI at T0 revealed the presence of two STs: three isolates belonged to ST101 and three to ST512. Regarding the resistance mechanisms to CAZ-AVI, two isolates carried a wild-type *bla*KPC-3 gene and four isolates a mutated *bla*KPC-3 (Table 1). Out of four mutated KPC-3 isolates, two (RM2.31.T0 and RM2.71.T0, both belonging to ST101) had a substitution at position 168 (P168L), defining KPC-46, while two isolates (RM2.10.T0 and RM2.15.T0, belonging to ST101 and ST512, respectively) had a substitution at position 179 (D179Y), defining KPC-31. The remaining isolates (RM2.05.T0 and RM2.21.T0) harbored *bla*KPC-3 with no substitutions and showed an MIC value for CAZ-AVI lower than that of isolates with a KPC variant (16/4 vs. 32/4 in isolates with KPC-46 and ≥64/4 in isolates with KPC-31) (Table 1).

Regarding the major porins that allow the diffusion of CAZ-AVI across the outer membrane (OmpK35 and OmpK36), all the isolates had truncated OmpK35 and mutated OmpK36 (Table 1). These isolates had mutations in *omp*K35, resulting in a truncated protein at amino acid 63 in ST101 isolates or at position 89 in ST512 isolates. OmpK36 had an insertion at position 134–135 of threonine and aspartic acid or glycine and aspartic acid.

Isolates from the same patient during the follow-up belonged to the same ST and harbored the same KPC from T0, except one case (patient 3), which lost KPC-31 and gained KPC-3. This isolate was susceptible to CAZ-AVI (Table 1). Notably, isolates with variant KPC-3 were susceptible to meropenem, while isolates with wild-type KPC-3 were resistant to meropenem. All ten isolates were susceptible to meropenem–vaborbactam (MVB) and to imipenem–relebactam (IMR) (Table 1).

The resistome analysis revealed the presence of several antibiotic resistance determinants in each isolate, along with the *bla*KPC gene (Table 2). Different antibiotic resistance genes were found by ST type. Genes coding for resistance to aminoglycoside, macrolide, lincosamide and streptogramin (MLS) were detected in all isolates, except one; sulphonamides, phenicols, rifampicin, tetracycline and trimethoprim/sulfamethoxazole resistance genes were identified in ST512 isolates only. Quinolone-related resistance gene mutations of *gy*r*A* and *parC* were found in all KPC-KP isolates. No tigecycline- or colistin-acquired resistance genes were found. One isolate that was resistant to colistin had a truncated MgrB protein (Supplementary Table S1).

Regarding the capsular biosynthesis locus (KL), isolates belonging to ST101 were associated with the KL17 locus and *wzi*-137 allele, while ST512 isolates were associated with KL107 and the *wzi*-154 allele (Table 3). The iron-scavenging siderophore yersiniabactin *ybt*9 gene cluster, associated with a mobile genetic element ICEKp3, was detected in all ST101 isolates, while, in ST512 isolates, no virulence genes were detected. In all KPC-KP isolates, the presence of several plasmids was observed: IncF was present in all isolates, IncR and Col440 in ST101 and IncX3 in ST512 isolates (Table 3). 

**Figure 2:**
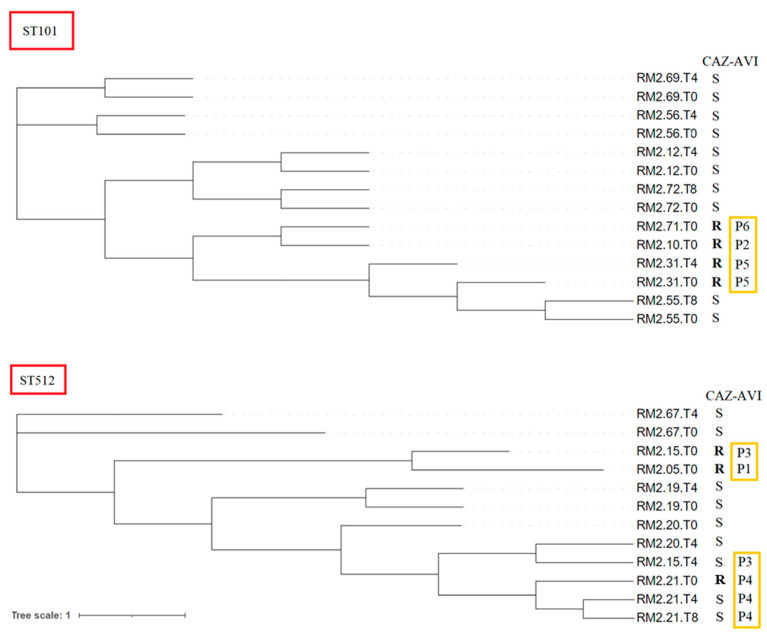
SNP-based core genome phylogeny of KPC-KP isolates from carriage in elderly patients, according to ST. Isolates’ IDs are indicated on the right of the tree. Isolates resistant to CAZ-AVI are marked in bold. Patients’ numbers are indicated on the right side of the figure, in yellow boxes. CAZ-AVI, ceftazidime–avibactam; R, resistant; S, susceptible.

Phylogenetic analysis based on core genome single-nucleotide polymorphisms (SNPs) of KPC-KP isolates resistant to CAZ-AVI compared to KPC-KP isolates from the intestinal carriage in other patients in the same neuro-rehabilitation structure, according to ST, showed that CAZ-AVI-resistant KPC-KP isolates clustered together (Figure 2). SNP analysis of CAZ-AVI-resistant KPC-KP isolates belonging to ST101 showed that patients 2, 5 and 6 had isolates closely related to each other (RM2.10.T0, RM2.31.T0, RM2.31.T4, RM2.71.T0, SNP range 7–11) (Supplementary Table S2). Regarding ST512 isolates, phylogenetic analysis of KCP-KP isolates resistant to CAZ-AVI (RM2.05.T0, RM2.15.T0, RM2.21.T0) showed that they were separated into two groups. Core genome SNP variation between KPC-KP isolates from the same patient (T0 versus latest follow-up times) showed that, for patient 4, the persistent isolates at the follow-up times appeared to be closely related to that obtained at enrolment, having two consecutive isolates with 4–7 SNPs from isolate at T0; patient 5 had a consecutive isolate with five SNPs. Of note, patient 3 had a consecutive isolate susceptible to CAZ-AVI (RM2.15.T4) that was more closely related to the susceptible isolate of another patient (RM2.20.T4), with only six SNPs, than to the CAZ-AVI-resistant isolate obtained at enrolment from the same patient (isolate RM2.15.T0 vs. RM2.15.T4, 75 SNPs) (Figure 2 and Supplementary Table S2).

## 3. Discussion

*K. pneumoniae* frequently shows resistance to carbapenems, considered “last-resort” drugs for the management of infection caused by this pathogen, together with other beta-lactam antibiotics. Recently, new antibiotics have been released and are active against CPE, including the β-lactam–β-lactamase inhibitor combinations [8,9]. Unfortunately, when they were introduced in clinical practice, resistance was soon reported [11], mainly due to mutations of the *bla*KPC-2 or *bla*KPC-3 genes, or by the overexpression of *bla*KPC along with outer membrane porin loss, bringing new challenges to clinical treatment [12,13]. Italy is an endemic country for KPC-*K. pneumoniae*; therefore, the presence of isolates resistant to the β-lactam–β-lactamase inhibitor combinations is alarming [19]. In a previous work on 156 invasive CR-KP in 2016, we found that ceftazidime–avibactam was one of the most active agents, with resistance rates of 2.6% [4]. In the present study, we analyzed KPC-*K. pneumoniae* isolates resistant to CAZ-AVI from colonized elderly patients at hospital discharge. These isolates were MDR and belonged to STs commonly circulating in Italy, ST101 and ST512. ST101 is one of the emerging high-risk clones causing hospital infections and outbreaks [20,21] and recently spread in Italy, contributing to changing the country’s epidemiology from the predominance of clonal group (CG) 258 toward a polyclonal CP-KP population [4]. ST512 is a well-known clone involved in the spread of antimicrobial resistance, belonging to CG258 [4,22]. Resistance to CAZ-AVI was associated mainly with mutant *bla*KPC-3 but also to different resistance mechanisms, since two isolates harboring the original *bla*KPC-3 were resistant to CAZ-AVI. All CAZ-AVI-resistant KP isolates produced a KPC enzyme; four isolates had a KPC variant enzyme, with substitutions within the Ω-loop (Arg164 to Asp179) in the active site of KPC, which has recently been associated with enhanced affinity to ceftazidime, possibly leading to reduced avibactam binding [23]. KPC-31 is one of the enzymes more frequently found associated with CAZ-AVI resistance, and it caused a hospital outbreak in Argentina [24,25]. KPC-46 was already found in Spain, associated with CAZ-AVI resistance in KP in a ST307 clone [26]. Two isolates had a wild-type KPC-3 but exhibited resistance to CAZ-AVI, along with mutated OmpK35 and OmpK36 porins; these modifications possibly restrict antibiotic entry and act in synergy with carbapenemase enzymes to increase the level of resistance [12]. Mutations in genes encoding porins may prevent the production of a functional porin in the case of OmpK35 or lead to the production of a narrow pore in the case of OmpK36, blocking the influx of antibiotics in the bacterial cell [27,28]. In this study, isolates with mutated KPC-3 had reduced MIC for meropenem, being susceptible as previously reported [26,29]. This could result in missing such isolates, which cannot be detected by phenotypic methods if ceftazidime–avibactam is not routinely tested during antimicrobial susceptibility testing. Such a challenge for diagnostic laboratories could lead to the spread of “hidden” *bla*KPC genes in nosocomial pathogens, causing both colonization and infection. For these reasons, it is necessary to test for CAZ-AVI resistance via phenotypic methods and to apply molecular tools—and, in the future, also WGS—to the routine diagnostic activity to correctly identify antibiotic-resistant pathogens.

During the follow-up, three patients maintained the KP colonization, but only one patient at T4 carried an isolate that was still resistant to CAZ-AVI, possibly due to CAZ-AVI treatment performed during the stay in the rehabilitation unit. Another patient carried *bla*KPC-3 isolates at different times that exhibited different levels of CAZ-AVI susceptibility, being resistant at T0 and susceptible at T4 and T8. This could suggest a different level of expression of the KPC enzyme that cannot be revealed by WGS analysis. In a previous paper, Di Pilato et al. found increased KPC production as the most common resistance mechanism in their collection of KP isolates resistant to CAZ-AVI [30]. These isolates harbored *bla*KPC-3, lacked functional OmpK35 and carried mutated OmpK36 and were associated with resistance to MVB, pointing out the possible step-wise development of resistance against novel BLICs, depending on the magnitude of the *bla*KPC gene dosage in a similar genetic background [30]. Our study had some limitations, as we did not investigate carbapenemase expression. Differently from Di Pilato’s study, we did not find resistance to MVB in KPC-KP isolates. Nevertheless, it is possible that multiple mechanisms are involved in resistance to BLICs, other than Ω-loop mutations of KPC-3, in isolates with altered or lacking porins, and further studies are required to assess KPC expression. Moreover, for some patients, the possibility of new acquisitions during hospitalization could not be ruled out, since some isolates obtained during follow-up were similar to isolates from other patients, suggesting the possibility of the circulation of resistant isolates in the hospital setting.

## 4. Materials and Methods

### 4.1. Bacterial Strain Identification and Antimicrobial Susceptibility Testing (AST) 

One hundred and twelve KPC-KP isolates were collected during the period of February 2018–January 2020 from the intestinal carriage of hospitalized patients aged ≥65 years enrolled at hospital discharge (T0), as described previously, using rectal swabs [16]. Rectal swabs were streaked onto chromID CARBA SMART agar plates (bioMerieux, Marcy l’Etoile, France) and incubated at 35 °C for 48 h. Identification to species level was performed by matrix-assisted laser desorption/ionization time-of-flight mass spectrometry (MALDI-TOF/MS) using a Microflex Biotyper^®^ LT (Bruker Daltonik GmbH, Bremen, Germany). Carbapenemase production was investigated using the agar tablet/disc diffusion method (KPC/MBL and OXA-48 Confirm Kit; ROSCO Diagnostica A/S, Taastrup, Denmark). Of these patients, six were colonized by CAZ-AVI-resistant KP; during the 12-month follow-up, four additional KPC-KP isolates were obtained from the same six patients and were included in the study. At the National Reference Laboratory (NRL) at Istituto Superiore di Sanità, all the isolates underwent further phenotypic and molecular characterizations. Antibiotic susceptibility testing (AST) was carried out by the reference broth microdilution method using lyophilized, custom microtitration plates (Merlin Diagnostika, Berlin, Germany), and by reference agar dilution for fosfomycin using an AD fosfomycin panel (Liofilchem, Roseto degli Abruzzi, Italy). Meropenem–vaborbactam and imipenem–relebactam were tested by the broth microdilution method using lyophilized, custom microtitration plates (Thermo Fisher Scientific, Rodano, Italy). *E. coli* ATCC 25922 was used as the quality control strain. The interpretative breakpoints were based on EUCAST, version 13.0 [31]. For tigecycline, results were interpreted using the *E. coli* breakpoints.

### 4.2. Whole-Genome Sequencing and In Silico Analysis 

All the 10 KPC-KP isolates obtained from patients colonized by KPC-KP resistant to CAZ-AVI (6 at enrolment and 4 during the follow-up) were analyzed by whole-genome sequencing (WGS). Genomic DNA was extracted from an overnight culture using the NucleoSpin DNA Extract Kit (Macherey-Nagel, Duren, Germany). WGS was performed using Ion Torrent technology (Life Technologies, Thermo Fisher Scientific, Waltham, MA, USA), according to the manufacturers’ instructions. De novo assembly of sequence reads was performed using the SPAdes 3.14 software through the ARIES Galaxy server (https://w3.iss.it/site/aries/ assessed on 14 February 2023). MLST analysis was carried out according to the *K. pneumoniae* MLST website scheme (https://bigsdb.pasteur.fr/cgi-bin/bigsdb/bigsdb.pl?db=pubmlst_klebsiella_seqdef&page=profiles assessed on 8 April 2023). Identification of resistance and virulence genes related to KPC-KP was performed by Kleborate through the ARIES Galaxy server. A core genome single-nucleotide polymorphism (SNPs) phylogeny was generated by CSI Phylogeny at the Center for Genomic Epidemiology (https://cge.cbs.dtu.dk/services/, accessed on 8 April 2023) [32], using the complete genome of *K. pneumoniae* (accession no. CP083000 for ST101 and NZ_CP015822 for ST512) as a reference, and compared to sequences of KPC-KP isolates from intestinal carriage in other patients in the same hospital according to STs isolated in our previous study [16]. The CSI Phylogeny tool calls SNPs, filters the SNPs, performs site validation and infers the phylogeny based on the concatenated alignment of the high-quality SNPs, through procedures that identify variations in whole-genome sequencing reads [33]. Briefly, reads were mapped to the reference sequence using BWA v. 0.7.2; SNPs were called using the mpileup part of SAMTools v. 0.1.18 and were filtered out if the depth at the SNP position was not at least 10×. All genome mappings were then compared and all positions where SNPs were called in at least one mapping were validated in all mappings. Positions where no SNPs were found or where SNPs had been ignored were assumed to be identical to the base in the reference sequence. A maximum likelihood tree was created from the alignment. The resulting maximum likelihood phylogenetic tree was visualized by uploading the Newick file to the Interactive Tree of Life platform, iTOL (http://itol.embl.de/upload.cgi, accessed on 8 April 2023). In silico analysis of the assembled contigs was also performed using tools available at the CGE server and screened for plasmid content using the PlasmidFinder tool. Plasmids of the IncF or IncA/C types were subtyped by assigning a replicon allele at the plasmid MLST site (https://pubmlst.org/plasmid/, accessed on 8 April 2023).

## 5. Conclusions

In conclusion, the presence of *K. pneumoniae* harboring KPC-3 variant enzymes, such as KPC-31 and KPC-46, or over-producing KPC, which confer resistance to CAZ-AVI, should be carefully monitored, by determining the phenotypic susceptibility of KP isolates to ceftazidime–avibactam, to prevent the missing of such isolates. This is essential so that timely infection control measures can be instituted because the spread of KP carrying variant KPC or over-producing KPC would seriously limit treatment alternatives for infections in healthcare and community settings.

## Figures and Tables

**Table 1:** Characteristics of ten KPC-KP isolates included in this study.

				Porins		MIC (mg/L)									
Patient	Isolate	ST	Carba	OmpK35 *	OmpK36	CAZ-AVI		MEM		MVB		IMI		IMR	
P1	RM2.05.T0	512	KPC-3	AA89 stop	GD ins	16/4	R	>64	R	1/8	S	>8	R	0.5/4	S
P2	RM2.10.T0	101	KPC-31	AA63 stop	TD ins	>64/4	R	2	S	0.25/8	S	≤1	S	0.12/4	S
P3	RM2.15.T0	512	KPC-31	AA89 stop	GD ins	64/4	R	2	S	1/8	S	≤1	S	0.12/4	S
	RM2.15.T4	512	KPC-3	AA89 stop	GD ins	8/4	S	>64	R	0.25/8	S	>8	R	0.25/4	S
P4	RM2.21.T0	512	KPC-3	AA89 stop	GD ins	16/4	R	>64	R	8/8	S	>8	R	0.25/4	S
	RM2.21.T4	512	KPC-3	AA89 stop	GD ins	4/4	S	32	R	≤0.06/8	S	4	I	0.12/4	S
	RM2.21.T8	512	KPC-3	AA89 stop	GD ins	2/4	S	>64	R	0.5/8	S	>8	R	0.12/4	S
P5	RM2.31.T0	101	KPC-46	AA63 stop	TD ins	32/4	R	1	S	0.25/8	S	≤1	S	0.12/4	S
	RM2.31.T4	101	KPC-46	AA63 stop	TD ins	64/4	R	1	S	0.25/8	S	≤1	S	0.12/4	S
P6	RM2.71.T0	101	KPC-46	AA63 stop	TD ins	32/4	R	1	S	0.25/8	S	≤1	S	0.12/4	S

* Truncated protein at amino acid position 89 or 63, respectively; GD, glycine 134–aspartic acid 135 duplication; TD, threonine 134–aspartic acid 135 duplication; MVB, meropenem–vaborbactam; IMR, imipenem–relebactam.

**Table 2:** Resistome analysis of ten KPC-KP isolates from carriage in elderly patients.

Patient	Isolate	ST	β-Lactamases	Aminoglycoside Modifying Enzyme	Fluoroquinolones	MLS	Phenicols	RIF	SUL	TET	TMT
P1	RM2.05.T0	512	KPC-3, TEM-1D, SHV-11	*aac(6′)-Ib*, *aadA2, aph(3′)-Ia*	GyrA-S83I, ParC-S80I	*mphA*	*catA1*	-	*sul1*	-	*dfrA12*
P2	RM2.10.T0	101	KPC-31, SHV-1	*armA*	GyrA-S83Y/D87N ParC-S80I	*mphE*, *msrE*	-	-	-	-	-
P3	RM2.15.T0	512	KPC-31, SHV-11	*aac(6′)-Ib’,aadA2*, *aph(3′)-Ia*	GyrA-S83I, ParC-S80I	*mphA*	*catA1*	*-*	*sul1*	*-*	*dfrA12*
	RM2.15.T4	512	KPC-3, SHV-11	*aac(6′)-Ib’*	GyrA-S83I, ParC-S80I	-	-	-	-	-	-
P4	RM2.21.T0	512	KPC-3, CMY-16, OXA-10, SHV11	*aadA1, aadA2*, *aac(6′)-Ib3, strA*	GyrA-S83I, ParC-S80I	*mphA*	*catA1*, *cmlA5*, *floR*	*arr2*	*sul1*, *sul2*	*tetA*	*dfrA12*, *dfrA14*
	RM2.21.T4	512	KPC-3, CMY-16, OXA-10, SHV-11, TEM-1D	*aadA1, aadA2*, *aac(6′)-Ib3,strA*	GyrA-83I, ParC-80I	-	*cmlA5*, *floR*	*arr2*	*sul1*, *sul2*	*tetA*	*dfrA14*
	RM2.21.T8	512	KPC-3, CMY-16, OXA-10, SHV-11, TEM-1D	*aadA1, aadA2*, *aac(6′)-Ib3, strA*	GyrA-83I, ParC-80I	-	*cmlA5*, *floR*	*arr2*	*sul1*, *sul2*	*tetA*	*dfrA14*
P5	RM2.31.T0	101	KPC-46, SHV-1	*armA*	GyrA-S83Y/D87N ParC-S80I	*mphE*, *msrE*	-	-	-	-	-
	RM2.31.T4	101	KPC-46, SHV-1	*armA*	GyrA-S83Y/D87N ParC-S80I	*mphE*, *msrE*	-	-	-	-	-
P6	RM2.71.T0	101	KPC-46, SHV-1	-	GyrA-S83Y/D87NParC-S80I	*mphE*, *msrE*	-	-	-	-	-

ST, sequence type; MLS, macrolide, lincosamide and streptogramin; RIF, rifampicin; SUL, sulphonamide; TET, tetracycline; TMT, trimethoprim.

**Table 3:** Virulome and plasmid analysis of ten KPC-KP isolates from carriage in elderly patients.

Patient	Isolate	ST	KLocus	Virulence Factor	Plasmid	pMLST
P1	RM2.05.T0	512	KL107 (*wzi*-154)	-	IncFIB, IncFII, IncX3,ColRNAI	IncF [K1:A-:B-]
P2	RM2.10.T0	101	KL17 (*wzi*-137)	*ybt*9/ ICEKp3	IncFIA, IncFIB, IncFII, IncR,Col440,ColRNAI	IncF [K1:A13:B-]
P3	RM2.15.T0	512	KL107 (*wzi*-154)	-	IncFIB, IncFII, IncX3	IncF [K2:A-:B-]
	RM2.15.T4	512	KL107 (*wzi*-154)	-	IncFIB, IncFII, IncX3, ColRNAI	IncF [K2:A-:B-]
P4	RM2.21.T0	512	KL107 (*wzi*-154)	-	IncFIB, IncFII, IncC IncX3	IncF [K2:A-:B-] IncC [C3]
	RM2.21.T4	512	KL107 (*wzi*-154)	-	IncFIB, IncFII, IncC IncX3	IncF [K2:A-:B-] IncC [C3]
	RM2.21.T8	512	KL107 (*wzi*-154)	-	IncFIB, IncFII, IncC IncX3	IncF [K2:A-:B-] IncC [C3]
P5	RM2.31.T0	101	KL17 (*wzi*-137)	*ybt*9/ ICEKp3	IncFIA, IncFIB, IncFII,IncR,Col440,ColRNAI	IncF [K2:A13:B-]
	RM2.31.T4	101	KL17 (*wzi*-137)	*ybt*9/ ICEKp3	IncFIA, IncFIB, IncFII,IncR,Col440,ColRNAI	IncF [K2:A13:B-]
P6	RM2.71.T0	101	KL17 (*wzi*-137)	*ybt*9/ ICEKp3	IncFIA, IncFIB, IncFII,IncR,Col440,ColRNAI	IncF [K2:A13:B-]

## Data Availability

Sequence data were submitted to GenBank at NCBI under the BioProjects PRJNA937920 and PRJNA739519.

## Supplementary Materials

The following supporting information can be downloaded at: https://www.mdpi.com/article/10.3390/antibiotics12081282/s1, Table S1: Antibiotic susceptibility of ten KPC-KP isolates included in this study; Table S2: Core genome single-nucleotide polymorphisms (SNPs) variations between *K. pneumoniae* isolates from the same patients (T0 versus latest follow-up times) and between each other.
