# Supplementary material for: Whole-Genome Sequencing and Molecular Analysis of Ceftazidime–Avibactam-Resistant KPC-Producing Klebsiella pneumoniae from Intestinal Colonization in Elderly Patients
Source: Antibiotics (Basel). 2023 Aug 3;12(8):1282. doi: 10.3390/antibiotics12081282 (PMC10451778; doi:10.3390/antibiotics12081282)
Supplement: Supplementary file 1 [file antibiotics-12-01282-s001.zip › Supplementary Table 1.pdf]

Supplementary Table 1. Antibiotic susceptibility of ten KPC-KP isolates included in this study

			MIC (mg/l)																																
Patient	Isolate	Date of isolation	**ERT***		**MEM**		**IMI**		**AMK**		**GEN**		**AMC**		**PT**		**FEP**		**CAZ**		**CAZ-AVI**		**CRO**		**CIP**		**LEV**		**TGC**		**COL**		**FOS**		**SXT**
1	RM2.05.T0	16/02/2018	>2	**R**	>64	**R**	>8	**R**	16	**R**	≤1	S	>64/2	**R**	>128/4	**R**	>16	**R**	>64	**R**	16/04	**R**	>4	**R**	>1	**R**	>8	**R**	0.5	S	>4	**R**	>256	**R**	>8/152
2	RM2.10.T0	22/08/2018	>2	**R**	2	S	≤1	S	>16	**R**	>8	**R**	16/2	**R**	8/4	S	>16	**R**	>64	**R**	>64/4	**R**	>4	**R**	>1	**R**	>8	**R**	≤0.25	S	1	S	16	S	≤1/19
3	RM2.15.T0	24/11/2018	>2	**R**	2	S	≤1	S	16	**R**	≤1	S	>64/2	**R**	>128/4	**R**	>16	**R**	>64	**R**	64/4	**R**	>4	**R**	>1	**R**	>8	**R**	0.5	S	≤0.5	S	>256	**R**	>8/152
	RM2.15.T4	05/04/2019	>2	**R**	>64	**R**	>8	**R**	>16	**R**	≤1	S	>64/2	**R**	>128/4	**R**	>16	**R**	>64	**R**	8/4	S	>4	**R**	>1	**R**	>8	**R**	0.5	S	≤0.5	S	64	**R**	≤1/19
4	RM2.21.T0	21/02/2019	>2	**R**	>64	**R**	>8	**R**	>16	**R**	4	**R**	>64/2	**R**	>128/4	**R**	>16	**R**	>64	**R**	16/4	**R**	>4	**R**	>1	**R**	>8	**R**	0.5	S	1	S	32	S	>8/152
	RM2.21.T4	21/06/2019	>2	**R**	32	**R**	4	I	>16	**R**	≤1	S	>64/2	**R**	>128/4	**R**	>16	**R**	>64	**R**	4/4	S	>4	**R**	>1	**R**	>8	**R**	0.5	S	≤0.5	S	8	S	>8/152
	RM2.21.T8	25/11/2019	>2	**R**	>64	**R**	>8	**R**	>16	**R**	>8	**R**	>64/2	**R**	>128/4	**R**	>16	**R**	>64	**R**	2/4	S	>4	**R**	>1	**R**	>8	**R**	0.5	S	1	S	32	S	>8/152
5	RM2.31.T0	22/05/2019	>2	**R**	1	S	≤1	S	>16	**R**	>8	**R**	16/2	**R**	16/4	**R**	16	**R**	>64	**R**	32/4	**R**	>4	**R**	>1	**R**	>8	**R**	≤0.25	S	≤0.5	S	>256	**R**	≤1/19
	RM2.31.T4	16/09/2019	2	**R**	1	S	≤1	S	>16	**R**	>8	**R**	32/2	**R**	16/4	**R**	16	**R**	>64	**R**	64/4	**R**	>4	**R**	>1	**R**	>8	**R**	≤0.25	S	≤0.5	S	>256	**R**	≤1/19
6	RM2.71.T0	30/01/2020	>2	**R**	1	S	≤1	S	>16	**R**	>8	**R**	16/2	**R**	16/4	**R**	>16	**R**	>64	**R**	32/4	**R**	>4	**R**	>1	**R**	>8	**R**	≤0.25	S	≤0.5	S	>256	**R**	≤1/19

*Resistant isolates are marked in bold; ERT, ertapenem; MEM, meropenem; IMI, imipenem; AMK, amikacin; GEN, gentamicin; AMC, amoxicillin/clavulanic; PT, piperacillin/tazobactam; FEP, cefepime; CAZ ceftazidime; CAZ-AVI, ceftazidime/avibactam; CRO, ceftriaxone; CIP, ciprofloxacin; LEV, levofloxacin; TGC, tigecycline; COL, colistin; FOS, fosfomycin; SXT trimethoprim-sulfamethoxazole.
