# Supplementary material for: Whole-Genome Sequencing and Molecular Analysis of Ceftazidime–Avibactam-Resistant KPC-Producing Klebsiella pneumoniae from Intestinal Colonization in Elderly Patients
Source: Antibiotics (Basel). 2023 Aug 3;12(8):1282. doi: 10.3390/antibiotics12081282 (PMC10451778; doi:10.3390/antibiotics12081282)
Supplement: Supplementary file 1 [file antibiotics-12-01282-s001.zip › Supplementary Table 2.pdf]

Supplementary Table 2. Core genome single nucleotide polymorphisms (SNPs) variation between *K. pneumoniae* isolates from the same patients (T0 versus latest follow up times) and between each other

ST101

	RM2.10.T0	RM2.12.T0	RM2.12.T4	RM2.31.T0	RM2.31.T4	RM2.55.T0	RM2.55.T8	RM2.56.T0	RM2.56.T4	RM2.69.T0	RM2.69.T4	RM2.71.T0	RM2.72.T0	RM2.72.T8
RM2.10.T0	0	10	10	7	6	7	9	14	18	11	10	11	12	16
RM2.12.T0	10	0	2	11	10	11	13	18	22	15	14	19	4	8
RM2.12.T4	10	2	0	11	10	11	13	18	22	13	14	19	4	8
RM2.31.T0	7	11	11	0	5	6	8	15	19	12	11	16	13	17
RM2.31.T4	6	10	10	5	0	5	7	14	18	11	10	13	12	12
RM2.55.T0	7	11	11	6	5	0	8	15	19	12	11	16	13	17
RM2.55.T8	9	13	13	8	7	8	0	17	21	14	13	18	15	19
RM2.56.T0	14	18	18	15	14	15	17	0	4	17	18	23	18	24
RM2.56.T4	18	22	22	19	18	19	21	4	0	21	22	27	22	28
RM2.69.T0	11	15	13	12	11	12	14	17	21	0	5	20	15	21
RM2.69.T4	10	14	14	11	10	11	13	18	22	5	0	19	16	20
RM2.71.T0	11	19	19	16	13	16	18	23	27	20	19	0	21	23
RM2.72.T0	12	4	4	13	12	13	15	18	22	15	16	21	0	8
RM2.72.T8	16	8	8	17	12	17	19	24	28	21	20	23	8	0

ST512

	RM2.05.T0	RM2.15.T0	RM2.15.T4	RM2.19.T0	RM2.19.T4	RM2.20.T0	RM2.20.T4	RM2.21.T0	RM2.21.T4	RM2.21.T8	RM2.67.T0	RM2.67.T4
RM2.05.T0	0	24	79	73	76	84	83	81	80	80	88	79
RM2.15.T0	24	0	75	69	72	80	79	77	76	76	84	75
RM2.15.T4	79	75	0	48	43	23	6	18	19	19	73	58
RM2.19.T0	73	69	48	0	9	53	52	50	49	49	67	58
RM2.19.T4	76	72	43	9	0	48	47	45	44	44	70	55
RM2.20.T0	84	80	23	53	48	0	27	25	26	26	78	63
RM2.20.T4	83	79	6	52	47	27	0	22	23	23	77	62
RM2.21.T0	81	77	18	50	45	25	22	0	4	7	75	60
RM2.21.T4	80	76	19	49	44	26	23	4	0	3	72	59
RM2.21.T8	80	76	19	49	44	26	23	7	3	0	72	59
RM2.67.T0	88	84	73	67	70	78	77	75	72	72	0	59
RM2.67.T4	79	75	58	58	55	63	62	60	59	59	59	0
